# Efgartigimod for generalized myasthenia gravis: a comprehensive review of clinical evidence and future perspectives

**DOI:** 10.3389/fneur.2026.1813230

**Published:** 2026-06-10

**Authors:** Lingyu Jiang, Jiaping Wei, Junqi Qin, Jing He, Shengjing Liang, Jianwei Huang, Yunzhi Ma, Yifan Zhou, Yonglong Zhong

**Affiliations:** 1 Department of Intensive Care Unit, The People's Hospital of Guangxi Zhuang Autonomous Region, Guangxi Academy of Medical Sciences, Nanning, China; 2 Department of Thoracic Surgery, The People’s Hospital of Guangxi Zhuang Autonomous Region, Guangxi Academy of Medical Sciences, Nanning, China

**Keywords:** biologic therapy, efgartigimod, FcRn inhibitor, myasthenia gravis, subcutaneous

## Abstract

Efgartigimod, a first-in-class neonatal Fc receptor (FcRn) antagonist, has transformed the therapeutic landscape of generalized myasthenia gravis (gMG). This review synthesizes evidence from pivotal trials to real-world applications. The phase 3 ADAPT trial demonstrated rapid, clinically meaningful improvements in Myasthenia Gravis Activities of Daily Living (MG-ADL) scores in anti-acetylcholine receptor antibody-positive (AChR-Ab+) patients, with a favorable safety profile. Long-term extension studies confirmed reproducible efficacy and sustained safety across multiple treatment cycles. Subsequent research optimized dosing through flexible, individualized regimens and a subcutaneous formulation, enhancing patient convenience. Extensive real-world evidence validates these findings across diverse populations, including antibody-negative patients, those with acute exacerbations or myasthenic crisis, and thymoma-associated MG, consistently showing rapid response and steroid-sparing effects. When real-world evidence is stratified by antibody status, thymic pathology, and age at onset, efgartigimod shows consistent benefits across most subgroups, including MuSK-positive and seronegative patients. Emerging data suggest immunomodulatory effects beyond IgG reduction, influencing B-cell populations and gene expression profiles. While comparative efficacy against other novel biologics (complement inhibitors, other FcRn antagonists) remains under investigation through indirect analyses, efgartigimod’s rapid onset, cyclical administration allowing drug holidays, and broad applicability distinguish it. Distinct biomarker profiles are emerging for FcRn blockade versus complement inhibition, with the former linked to measurable changes in IgG subclasses and immune cell phenotypes. Ongoing research focuses on optimizing patient selection, exploring combination therapies, and expanding applications to other IgG-mediated autoimmune diseases. Efgartigimod represents a cornerstone of targeted immunotherapy in MG, offering personalized, effective, and well-tolerated disease management.

## Introduction

Myasthenia gravis (MG) is a chronic autoimmune disorder characterized by pathogenic immunoglobulin G (IgG) autoantibodies that disrupt neuromuscular transmission, leading to fatigable muscle weakness (1). While conventional immunosuppressive therapies remain foundational, a substantial proportion of patients, estimated around 20%, experience refractory disease or intolerable side effects, highlighting a significant unmet need (2). The therapeutic landscape for generalized MG (gMG) has been transformed in recent years by the advent of targeted biologics. These novel agents offer mechanisms of action directed at specific components of the immune pathogenesis, promising faster onset, improved efficacy, and potentially better safety profiles compared to broad immunosuppressants (3, 4).

Among these breakthroughs, modulation of the neonatal Fc receptor (FcRn) has emerged as a pivotal strategy. FcRn plays a crucial physiological role in extending the half-life of IgG by rescuing it from lysosomal degradation and recycling it back into circulation (5). By competitively inhibiting FcRn, therapeutic agents can accelerate the catabolism of all IgG subclasses, including pathogenic autoantibodies, without affecting other immunoglobulin isotypes. Efgartigimod, a human IgG1-derived Fc fragment engineered for high-affinity binding to FcRn, represents the first-in-class agent utilizing this mechanism (6, 7). Following its initial approval in the United States in December 2021 for anti-acetylcholine receptor antibody-positive (AChR-Ab+) gMG, it has gained regulatory approval in numerous other regions, including Japan and the European Union (8).

The development of efgartigimod signifies a paradigm shift toward precision immunomodulation in MG. Its approval was grounded in robust phase 3 trial data, and subsequent integration into clinical practice has prompted extensive real-world investigation. Current research extends beyond confirming initial efficacy to exploring optimal dosing paradigms, comparative effectiveness against other biologics, utility in specific patient subsets and acute settings, long-term safety, and potential immunomodulatory effects beyond IgG reduction. This review synthesizes the substantial and rapidly evolving body of evidence on efgartigimod, from pivotal trials to real-world applications and future directions, providing a comprehensive overview of its role in the modern management of myasthenia gravis.

### Efficacy and safety of efgartigimod

The efficacy and safety profile of intravenous (IV) efgartigimod was established in the landmark phase 3 ADAPT trial, a randomized, placebo-controlled study (9). This trial enrolled patients with gMG, including both AChR-Ab+ and AChR-Ab- individuals, who had a Myasthenia Gravis Activities of Daily Living (MG-ADL) score of at least 5. Participants received four once-weekly infusions of efgartigimod (10 mg/kg) or placebo per treatment cycle. The primary endpoint, the proportion of AChR-Ab+ patients who were MG-ADL responders (defined as a ≥2-point improvement sustained for ≥4 weeks) in the first cycle, was met decisively. Sixty-eight percent of efgartigimod-treated AChR-Ab+ patients achieved this response, compared to 30% in the placebo group, corresponding to an odds ratio of 4.95. Improvements were rapid, often observed within 1 week of the first infusion, and paralleled a profound reduction in total IgG and AChR-Ab levels.

The clinical benefits observed in ADAPT were robust across multiple assessment scales. Significant and clinically meaningful improvements were also recorded in the Quantitative Myasthenia Gravis (QMG) score, the Myasthenia Gravis Composite (MGC) scale, and patient-reported outcomes such as the revised 15-item Myasthenia Gravis Quality of Life (MG-QoL15r) scale (10, 11). The correlation between the reduction in pathogenic IgG autoantibodies and clinical improvement provided strong proof of concept for the FcRn inhibition mechanism (10). Regarding safety, efgartigimod was generally well-tolerated. The most frequent treatment-emergent adverse events were headache and nasopharyngitis, with incidences similar to the placebo group. The rate of serious adverse events was low (5% vs. 8% for placebo), and no deaths were reported during the trial (9).

The long-term durability and reproducibility of efgartigimod’s effects were evaluated in the open-label extension study, ADAPT+ (12). Patients who rolled over from ADAPT could receive repeated, individualized treatment cycles based on clinical evaluation. Interim results demonstrated that clinically meaningful improvements in MG-ADL and QMG scores were reproducible across multiple treatment cycles, with some participants receiving up to 17 cycles. Over 217.6 patient-years of exposure, the safety profile remained consistent, with no new safety signals identified. Headache, COVID-19, and nasopharyngitis were the most commonly reported adverse events. This extension study supported the long-term safety, tolerability, and efficacy of efgartigimod with chronic, cyclical use (7, 12).

Further supporting its clinical utility, post-hoc analyses of ADAPT data revealed that efgartigimod’s benefits extended across all muscle group subdomains. Significant improvements were noted in bulbar, ocular, limb/gross motor, and respiratory functions as captured by both MG-ADL and QMG sub-items (13). Moreover, these clinical gains translated into meaningful improvements in health-related quality of life and health utility values, underscoring the comprehensive patient benefit beyond symptom scores (11, 14). The collective evidence from the pivotal ADAPT program firmly established efgartigimod as an effective, rapid-acting, and generally well-tolerated therapy for AChR-Ab+ gMG.

### Optimization of dosing regimens

The initial ADAPT protocol introduced an individualized, response-driven dosing model, where subsequent treatment cycles were initiated based on clinical evaluation rather than a fixed schedule. This flexibility acknowledged the variable duration of therapeutic effect among patients. Subsequent research has focused on refining and expanding dosing options to enhance convenience and optimize clinical outcomes. The phase 3b ADAPT NXT study directly compared two structured approaches: fixed cycles (three cycles of four weekly infusions with 4 weeks between cycles) versus an every-other-week (Q2W) regimen following an initial cycle (15). Both arms demonstrated rapid, robust, and sustained clinically meaningful improvement in MG-ADL scores over 21 weeks, with no significant difference in efficacy between the strategies. This study provided clinicians with evidence-supported alternative dosing frameworks.

In parallel, the development of a subcutaneous (SC) formulation of efgartigimod co-formulated with recombinant human hyaluronidase PH20 (efgartigimod PH20 SC) addressed a key patient need for a more convenient administration route. The phase 3 ADAPT-SC study was designed to demonstrate non-inferiority of the SC formulation to the IV formulation (16). The primary endpoint was the percentage reduction in total IgG at week 4. The study successfully met its endpoint, confirming that efgartigimod PH20 SC 1,000 mg was non-inferior to efgartigimod IV 10 mg/kg in reducing IgG levels. Clinically meaningful improvements in MG-ADL and QMG scores were observed as early as 1 week after the first SC administration, with maximal effects at week 4, mirroring the pharmacodynamic and clinical profile of the IV formulation.

The open-label extension ADAPT-SC+ further confirmed the long-term safety, tolerability, and reproducible efficacy of the SC formulation across multiple treatment cycles (16, 17). The availability of a self-injectable or caregiver-administered option improves treatment convenience, reduces the burden of clinic visits, and may facilitate broader access to therapy (17, 18). Real-world experiences have shown successful transitions from IV to SC formulations without loss of efficacy (18). The pharmacokinetic and pharmacodynamic profiles of both IV and SC efgartigimod have also been validated in Chinese populations, showing consistency with global data (19). Together, these advancements in dosing—encompassing flexible cycling, fixed-interval regimens, and a convenient SC option—provide a toolkit for personalized treatment, enabling clinicians to tailor therapy to individual patient lifestyles and disease dynamics.

### Real-world effectiveness and validation in diverse clinical settings

While randomized controlled trials provide high-quality evidence of efficacy under controlled conditions, real-world studies are essential to validate these findings in heterogeneous patient populations encountered in clinical practice. Numerous observational studies across different regions have consistently affirmed the effectiveness and safety of efgartigimod. These studies often include patients with more complex comorbidities, refractory disease, or those who would have been excluded from stringent clinical trials.

A large multicenter cohort study from China involving 61 patients, many with thymoma-associated MG (TAMG) or acute exacerbations, demonstrated rapid clinically meaningful improvement in 97% of patients within an average of 1.3 weeks (20). Similarly, real-world experiences from the United Kingdom, the United States, Italy, and Japan have reported significant reductions in MG-ADL scores, with responder rates ranging from 72% to over 80% after the first treatment cycle (21–23). These studies confirm the rapid onset of action seen in clinical trials. The treatment has also shown a notable steroid-sparing effect, allowing for reduction or discontinuation of concomitant corticosteroids in a substantial proportion of patients (24–26).

Real-world evidence has been particularly valuable in assessing efgartigimod in broader patient groups. For instance, in the Japanese post-marketing surveillance interim analysis, efgartigimod improved MG-ADL scores significantly regardless of antibody status (AChR-Ab+, MuSK-Ab+, or seronegative) (27). Furthermore, real-world analyses have begun to identify patterns of response and potential predictors. Shorter disease duration and more severe bulbar symptoms at baseline have been associated with an earlier and more robust response to the first infusion (28). Conversely, the presence of certain comorbidities like thymoma, other tumors, or additional autoimmune diseases may be associated with a less optimal response in some patients (29). This growing body of real-world data solidifies the role of efgartigimod as a mainstay therapy for gMG and provides practical insights for patient management.

### Comparative efficacy and positioning among novel biologics

The therapeutic arsenal for gMG now includes several novel biologics with distinct mechanisms, primarily complement inhibitors (e.g., eculizumab, ravulizumab, zilucoplan) and FcRn antagonists (e.g., efgartigimod, rozanolixizumab, batoclimab) (30, 31). Direct head-to-head trials are lacking, necessitating indirect comparisons and network meta-analyses to inform treatment choices. An indirect treatment comparison using data from the ADAPT (efgartigimod) and CHAMPION MG (ravulizumab) trials suggested that efgartigimod may provide faster and greater improvements in quality of life (MG-QoL15r) over 26 weeks, along with more rapid improvements in MG-ADL and QMG scores (32). However, the cumulative clinical benefit over 26 weeks, measured by area under the curve, was not significantly different for MG-ADL and QMG.

Network meta-analyses have attempted to rank these novel agents. One systematic review and meta-analysis found that both complement inhibitors and FcRn blockers were effective, with FcRn treatments showing a greater effect on QMG score reduction in the short term (33). Another Bayesian network meta-analysis ranked batoclimab, eculizumab, and zilucoplan highly for efficacy, while efgartigimod and rozanolixizumab were highlighted for their effectiveness, albeit with some agents like rozanolixizumab associated with a higher incidence of adverse events (34). A separate analysis focusing on benefit–risk and economic value suggested efgartigimod had a favorable profile, with the lowest number needed to treat for key efficacy outcomes and cost per improved outcome among the comparators assessed (35). A cost-effectiveness analysis also suggested that while both eculizumab and efgartigimod exceed common cost-effectiveness thresholds, they offer valuable rapid-acting options for refractory gMG (36).

Real-world comparative studies offer another perspective. A retrospective German multicenter study comparing complement inhibition (C5IT) with efgartigimod found comparable efficacy and safety between the two strategies over the first 6 months (30). Furthermore, a U.S. claims database analysis indicated that patients continuously treated with complement inhibitors (eculizumab/ravulizumab) achieved greater reductions in concomitant oral corticosteroid and nonsteroidal immunosuppressant use over 12 months compared to those treated with efgartigimod (37). These comparative insights, though not definitive, help clinicians position efgartigimod within the treatment algorithm. Its rapid onset, cyclical administration allowing for drug holidays, and efficacy across antibody subtypes (in some regions) are distinguishing features. The choice between FcRn antagonism and complement inhibition may therefore be influenced by factors such as desired speed of onset, preference for continuous versus intermittent therapy, antibody profile, and individual patient response.

### Application in specific populations and challenging scenarios

The utility of efgartigimod extends beyond the typical refractory gMG patient, showing promise in several specific and challenging clinical scenarios. To better guide clinical decision-making, available data can be grouped according to antibody profile, thymic pathology, and disease onset or course.

### Subgroups defined by antibody status

Although initially approved for AChR-Ab+ gMG, real-world studies in Japan and other regions where the label is broader have shown efficacy in patients with MuSK antibodies and in double-seronegative individuals (23, 27). For MuSK-Ab+ gMG, which is known for its poor response to acetylcholinesterase inhibitors and sometimes resistance to conventional immunosuppression, consistent improvements have been documented after efgartigimod treatment (38). In double-seronegative and triple-seronegative gMG, open-label and retrospective studies have reported clinically meaningful improvements, suggesting that FcRn inhibition may be beneficial even when conventional antibodies are undetectable (39).

### Subgroups defined by thymic pathology

A complex population is patients with thymoma-associated MG (TAMG), who often have severe and fluctuating symptoms. Perioperative management for thymectomy is particularly challenging due to the risk of postoperative crisis. Prospective and retrospective studies have shown that efgartigimod can rapidly optimize neurological status before surgery and maintain stability during the postoperative period, potentially reducing the incidence of myasthenic crisis and shortening hospital stays (40–42). In a dedicated phase 2 trial, perioperative efgartigimod led to significant symptom relief and safe surgical outcomes in patients with AChR-Ab+ TAMG (40). Data on non-thymomatous gMG (e.g., thymic hyperplasia or post-thymectomy) are largely captured within the broader phase 3 and real-world cohorts (9, 12, 20, 27).

### Subgroups defined by age at onset and disease course

Research has also explored efficacy in very-late-onset MG (VLOMG) and elderly patients (≥80 years), populations where treatment tolerance and comorbidity interactions are major concerns. Using the quantitative criteria for minimal manifestations or better status (MM-or-better) established in a large Japanese cohort of 3,800 MG patients—which defined strict MM-or-better as MG-ADL ≤ 2 (12)—several real-world studies reported good tolerability and significant clinical improvement in these age groups (43, 44). For acute exacerbations and myasthenic crisis (MC), where rapid reduction of pathogenic antibodies is lifesaving, numerous case series and small cohort studies have described successful outcomes with efgartigimod as add-on rescue therapy, facilitating rapid weaning from mechanical ventilation and preventing crisis progression (45–49). In a retrospective comparison with intravenous immunoglobulin (IVIg) for impending crisis, efgartigimod showed a greater reduction in MG-ADL scores at weeks 1 and 4 (50).

These findings collectively underscore the broad potential applicability of efgartigimod across the diverse spectrum of myasthenia gravis presentations.

### Long-term management, immunomodulatory effects, and steroid-sparing potential

The long-term management of gMG with efgartigimod is characterized by its cyclical, response-guided administration. Data from ADAPT+ and real-world studies indicate that patients require a mean of approximately 4–5 treatment cycles per year, though inter-cycle intervals vary widely based on individual disease dynamics (12, 27, 51). This individualized approach allows for sustained disease control while minimizing overall drug exposure. A significant and highly valued outcome of long-term efgartigimod therapy is its corticosteroid-sparing effect. Multiple studies have reported substantial reductions in daily prednisone or prednisolone doses following efgartigimod initiation (24, 25, 52). In a large U. S. claims analysis, patients on chronic glucocorticoids who started efgartigimod achieved a significant and progressive reduction in their average daily steroid dose over 12 months, with 66% of patients reducing their dose and 42% reaching a dose of ≤5 mg/day by 1 year (26). This ability to reduce or discontinue steroids mitigates the risk of long-term adverse effects.

Emerging research suggests that the therapeutic mechanism of efgartigimod may extend beyond simple IgG catabolism to include broader immunomodulatory effects. FcRn is expressed on various immune cells, including B cells and monocytes. Investigative studies have observed that efgartigimod treatment is associated with dynamic changes in peripheral immune cell populations, as detailed in the biomarker section below. These findings imply that FcRn blockade might exert a modulating effect on the autoimmune response. While the clinical significance of these immunological shifts is still being elucidated, they hint at a more complex mechanism of action that could contribute to sustained clinical benefit.

### Emerging biomarkers: FcRn blockade versus complement inhibition

Detailed analyses of peripheral immune cells have revealed reproducible changes following efgartigimod treatment. Increases in circulating memory B cells and plasma cells, coupled with specific gene expression profiles (e.g., upregulation of CD38, LAG3), have been reported, suggesting the drug may influence B-cell differentiation (53). Another study noted a transient increase in regulatory T cells and plasmablasts alongside a decrease in non-classical monocytes and Th17.1 cells during treatment (54). Proteomic analyses have suggested candidate molecules such as FGF-19 as potential response-related biomarkers (55). Together, these observations indicate that efgartigimod produces measurable pharmacodynamic biomarker shifts beyond total IgG reduction. By contrast, complement inhibitors (eculizumab, ravulizumab) are currently monitored using classical complement pathway activity (e.g., CH50) or soluble C5b-9 (sMAC) levels, but to date no robust predictive biomarker of clinical response has been validated for this class.

Comparing the two classes, FcRn blockade offers a clearer link to measurable changes in IgG subclasses and immune cell phenotypes, and preliminary predictive models have been proposed (56). Reliable response biomarkers for complement inhibitors remain a gap in the field. Future research should aim at developing a cross-mechanism biomarker panel to guide personalized selection between FcRn antagonists and complement-targeted therapies.

### Safety and tolerability profile

The safety profile of efgartigimod, established in clinical trials, has been largely corroborated by extensive real-world experience and post-marketing surveillance. The most commonly reported adverse events are generally mild to moderate in severity. With the intravenous formulation, headache and infections (such as nasopharyngitis, upper respiratory tract infections, and urinary tract infections) are frequently observed (9, 12, 22). The subcutaneous formulation introduces injection-site reactions as an expected and common adverse event, while maintaining a similar overall safety profile to the IV form (16).

Post-marketing pharmacovigilance analyses based on the FDA Adverse Event Reporting System (FAERS) database provide a broader view of potential safety signals. These analyses confirm common events like urinary tract infection and headache but have also identified signals for other conditions, including falls, nephrolithiasis, atrial fibrillation, and reports of bulbar palsy or symptom recurrence (57, 58). It is crucial to interpret these signals with caution, as disproportionality analysis indicates a statistical association but does not prove causation; many reported events could be related to the underlying disease or comorbidities. The median time to onset of adverse events has been reported between 57 to 101 days post-initiation (55, 56). Serious adverse events, including serious infections, are reported but appear infrequently. There have been reports of severe infections, including a case of Kaposi’s varicelliform eruption (eczema herpeticum) related to herpes simplex virus reactivation (59). However, treatment discontinuation due to adverse events is uncommon in both trials and real-world settings.

A specific consideration with FcRn antagonists is their effect on protective antibody titers. Studies have shown that efgartigimod reduces levels of vaccine-induced antibodies (e.g., against tetanus, varicella) to a similar extent as total IgG, though titers often remain above protective thresholds (60). Animal model data further demonstrate that FcRn antagonism does not impair the generation of vaccine-induced protective humoral and cellular responses or protection against viral challenges (59). Importantly, patients treated with efgartigimod retain the capacity to mount a functional IgG immune response to new vaccinations, such as those for influenza, pneumococcus, or COVID-19, although the peak titers achieved may be transiently lower (61). This indicates that while monitoring for infection is prudent, FcRn blockade does not cause broad immunosuppression. Overall, the safety and tolerability profile of efgartigimod is considered favorable, supporting its chronic use in the management of gMG, with routine clinical vigilance recommended for infections and other potential side effects.

## Conclusion and future perspectives

Efgartigimod has unequivocally established itself as a transformative therapy in the landscape of myasthenia gravis. From its foundation in the pivotal ADAPT trial to its validation in diverse real-world settings, the evidence consistently demonstrates rapid, clinically meaningful, and reproducible efficacy in reducing disease burden for a wide spectrum of patients with gMG. The development of a subcutaneous formulation and the exploration of flexible dosing regimens have enhanced treatment convenience, moving toward more personalized disease management. Its steroid-sparing capability addresses a critical long-term treatment goal, while its generally favorable safety profile permits sustained use. Stratification by antibody subtype, thymic pathology, and disease course in real-world studies has reinforced its broad utility across diverse clinical scenarios, from MuSK-positive and seronegative MG to thymoma-associated disease and myasthenic crisis.

Looking forward, several key research avenues will shape the future application of efgartigimod and FcRn inhibition. A major focus is on refining patient selection and predicting response. The identification of reliable biomarkers remains a priority; compared with complement inhibitors, where predictive markers are still lacking, FcRn blockade offers more direct pharmacodynamic readouts such as IgG subclass levels and immune cell phenotyping (53, 54, 56). Comparative biomarker studies across drug classes are urgently needed to guide individualized therapy. Efforts are underway to identify clinical or serological biomarkers that can forecast which patients will derive the greatest or most sustained benefit from therapy (56). The investigation into its immunomodulatory effects beyond IgG clearance may yield novel biomarkers and deepen the understanding of its mechanism (53, 55). Furthermore, its utility continues to be explored in challenging scenarios, such as acute crises, perioperative care, and overlapping autoimmune syndromes, where rapid antibody reduction is paramount (42, 45, 62).

The role of efgartigimod in treatment sequences and combinations is another area of active inquiry. Studies are examining its use as a bridging therapy to achieve rapid control before slower-acting oral immunosuppressants take effect, or in combination with other biologics like B-cell depleting agents for synergistic effect (62–64). Finally, the success of FcRn antagonism in MG has paved the way for its investigation in other IgG-mediated autoimmune diseases, such as chronic inflammatory demyelinating polyneuropathy, immune thrombocytopenia, and autoimmune encephalitis, potentially expanding its therapeutic impact (65). In conclusion, efgartigimod represents a cornerstone of targeted immunotherapy in MG. Ongoing and future research will continue to optimize its use, solidify its position within treatment algorithms, and explore its full potential in neurology and autoimmunity.
